# Cerebral venous thrombosis in patients with autoimmune disease, hematonosis or coronavirus disease 2019: Many familiar faces and some strangers

**DOI:** 10.1111/cns.14321

**Published:** 2023-06-27

**Authors:** Yifan Zhou, Huimin Jiang, Huimin Wei, Xuechun Xiao, Lu Liu, Xunming Ji, Chen Zhou

**Affiliations:** ^1^ Laboratory of Brain Disorders, Ministry of Science and Technology, Collaborative Innovation Center for Brain Disorders, Beijing Institute of Brain Disorders, Beijing Advanced Innovation Center for Big Data‐based Precision Medicine Capital Medical University Beijing China; ^2^ Beijing Advanced Innovation Center for Big Data‐Based Precision Medicine, School of Engineering Medicine Beihang University Beijing China; ^3^ Department of Neurology, Xuanwu Hospital Capital Medical University Beijing China; ^4^ Department of Neurosurgery, Xuanwu Hospital Capital Medical University Beijing China

**Keywords:** autoimmune diseases, blood diseases, COVID‐19, stroke, vector‐based vaccine, venous stroke

## Abstract

**Background:**

Cerebral venous thrombosis, a rare stroke, is characterized by neurological dysfunction caused by bleeding and/or infarction resulting from venous sinus thrombosis, the so‐called venous stroke. Current guidelines recommend anticoagulants as first‐line therapy in the treatment of venous stroke. With complicated causes of cerebral venous thrombosis, treatment is difficult, especially when combined with autoimmune diseases, blood diseases, and even COVID‐19.

**Aims:**

This review summarizes the pathophysiological mechanisms, epidemiology, diagnosis, treatment, and clinical prognosis of cerebral venous thrombosis combined with autoimmune diseases, blood diseases, or infectious diseases such as COVID‐19.

**Conclusion:**

A systematic understanding of particular risk factors that should not be neglected when unconventional cerebral venous thrombosis occurs and for a scientific understanding of pathophysiological mechanisms, clinical diagnosis, and treatment, thus contributing to knowledge on special types of venous stroke.

## Abstract 2

Cerebral venous thrombosis, also known as venous stroke, is a complicated subtype. some important risk factors, such as autoimmune diseases, hematological disorders, and even COVID‐19, are nonnegligible for venous stroke, while the specific pathologies of particular risk factors associated with venous stroke have yet to be studied. This review summarizes the pathophysiological mechanisms, epidemiology, diagnosis, treatment, and clinical prognosis of cerebral venous thrombosis combined with specific risk factors.

## 1 INTRODUCTION

Cerebral venous thrombosis (CVT), including thrombosis of the cerebral veins and dural sinuses, is a peculiar cerebrovascular disorder of current interest, partly because of the significant morbidity associated with disorder.^1^, ^2^, ^3^ CVT is a complicated stroke subtype, accounting for 0.5%–1% of all strokes, with a median diagnostic delay of 7 days, and the rate of missed diagnosis is 73%.^4^, ^5^, ^6^ Thus, CVT is a special stroke subtype that should not be ignored.

Anticoagulation therapy, such as subcutaneous low‐molecular‐weight heparin (LMWH) or new oral anticoagulants, has been regarded as the first‐line treatment for CVT.^7^ Patients with CVT who receive substandard anticoagulant therapy often have a good outcome.^8^, ^9^ However, at least 13% of all patients with CVT die or become severely handicapped.^10^, ^11^

Previous studies have identified blood stasis, endothelial cell damage or damage to the vessel wall, and hypercoagulation (Virchow's triad) as the three main elements of thrombosis.^12^ At present, it is well established that changes in the Virchow's triad can lead to the occurrence of CVT. For instance, endothelial cell damage in which collagen fibers under the endothelium are exposed activates platelets and coagulation factor XII and initiates the endogenous coagulation system; simultaneously, the damaged endothelial cells release a large amount of tissue factor, which activates coagulation factor VII and can also activate the exogenous coagulation system to induce the formation and even recurrence of thrombus.^10^, ^13^ Therefore, the combination of anticoagulation and etiological treatment is the key therapy, but the risk factors and causes of CVT are complicated and diverse, such as sex‐specific factors (use of estrogen‐progesterone, puerperium, oral contraceptives, pregnancy), hereditary thrombophilia factors (protein C, S or antithrombin III deficiency), acquired thrombophilia factors (antiphospholipid syndrome, nephrotic syndrome, hyperhomocysteinemia), and other risk factors (anemia, infections).^14^ These diverse causative factors/etiologies pose a challenge for the clinical management of CVT, especially when combined with autoimmune diseases, hematological disorders, and even COVID‐19.^15^, ^16^, ^17^ Thus, the purpose of this study was to review the pathophysiological mechanisms and clinical features of special types of venous stroke and to provide guidance for further research on the pathophysiological mechanisms, clinical diagnosis, and treatment of special types of venous stroke.

## 2 VENOUS STROKE IN PATIENTS WITH AUTOIMMUNE DISEASES

### 2.1 Venous stroke in patients with Behçet's disease

Behçet's disease (BD) is a systemic variable vessel vasculitis with an unknown etiology that involves the venous and arterial systems.^18^, ^19^ BD is one of the leading causes of venous stroke in Mediterranean and Middle Eastern countries.^20^ The pathogenesis of CVT in BD is not fully clear.^21^ The likely cause of CVT in BD patients is venous inflammation‐induced endothelial dysfunction and abnormal activation of coagulation function.^22^ Assessment of a multicenter cohort from the United States showed that the proportion of venous stroke patients with BD was approximately 1%.^11^ Furthermore, an international study on cerebral vein and dural sinus thrombosis (ISCVT) achieved the same results.^10^ BD patients with venous stroke tended to be younger and predominantly male.^23^

Thrombosis was detected mostly in the transverse sinus in BD complicated with venous stroke.^24^ In a retrospective case–control study of 840 BD patients, 21 were diagnosed with venous stroke, as reported by Shi et al.,^22^ and the transverse sinus was found to be the most frequently affected site, followed by the superior sagittal sinus, sigmoid sinus, and straight sinus. Venous stroke‐BD patients typically present with vasculitis such as trilogy of BD and intracranial hypertension syndrome, with headache being the primary symptom.^25^ In the VENOST study, 1144 patients with venous stroke were enrolled from 35 centers, and 9.4% of patients were shown to have BD.^26^ Headache accounted for 92.6% of the reported symptoms, followed by visual field defects (38%), nausea or vomiting (25.9%), cranial nerve palsy (14.8%), focal neurologic deficits (8.73%), epileptic seizures (7.4%) and altered consciousness (4.6%). Symptom onset in venous stroke‐BD patients corresponded to the subacute form.^27^

Venous inflammation‐induced endothelial dysfunction and abnormal activation of coagulation function may be the mechanism of thrombosis. Systemic anticoagulation is the standard treatment for venous stroke.^14^ Anticoagulation therapy is a recommended treatment for venous stroke, involving the intravenous administration of heparin or LMWH.^14^, ^28^ Following the acute phase of venous stroke, a minimum duration of 3–6 months of warfarin administration with a target international normalized ratio (INR) range of 2–3 is advised.^14^, ^29^ In cases where the patient exhibits intolerance to warfarin, alternative oral anticoagulants, such as rivaroxaban and dabigatran, may be deemed as viable options.^30^ Based on a cohort of 64 patients with BD‐associated venous stroke, Saadoun et al.^31^ concluded that compared to anticoagulation alone, the addition of immunosuppressive therapy did not affect either the neurological outcome or thrombosis recurrence risk. To date, there has yet to be a consensus on whether a combined treatment of immunosuppressive agents and anticoagulation is preferable to anticoagulation or immunosuppressive therapy alone for venous stroke in BD patients.^20^ Endovascular treatment (EVT) should be considered for patients with neurological deterioration despite anticoagulation or sever venous stroke, presenting with worsening neurological symptoms, cortical venous outflow stasis, intracerebral hemorrhage, deep venous thrombosis, worsening seizures and coma.^32^, ^33^ Although the TO‐ACT trial did not demonstrate a significant improvement in functional outcome for patients with venous stroke who underwent EVT with standard medical care, some studies suggest that patients with severe venous stroke may benefit from EVT.^34^, ^35^ The VENOST study demonstrated that the functional outcome of venous stroke in patients with BD was positive; only 12% of patients who presented with altered consciousness and cranial nerve involvement had poor outcomes (mRS ≥ 2).^27^

### 2.2 Venous stroke in patients with systemic lupus erythematosus

Systemic lupus erythematosus (SLE) is a chronic autoimmune disease that can exhibit heterogeneous clinical manifestations.^36^ The central nervous system (CNS) is one of the major organs affected by SLE, termed neuropsychiatric SLE.^37^ Venous stroke is one of the complications accompanying SLE.^38^ Vasculitis associated with endothelial cell injury mediated by immune‐complex deposition has been proposed as the primary pathogenesis of venous stroke in patients with SLE.^39^ The hypercoagulable state and correlation with the presence of anti‐phospholipid antibodies attributed to thrombosis in SLE‐related venous stroke could be another pathogenesis.^39^ Venous stroke is extremely rare in patients with SLE. In a retrospective, single‐center study in China of 4747 hospitalized SLE patients, 17 had a venous stroke, and among 17 SLE patients with venous stroke, 70.6% were women, with a median age of 30.^40^ Among the 1144 patients with venous stroke in the VENOST cohort, 15 had SLE, 86.7% were female, and the mean age was 39.9 years.^26^ Overall, SLE patients with venous stroke tended to be younger and predominantly female.

The transverse sinus (82.4%) was the most frequent location of the thrombus, and the sagittal sinus (35.3%) and sigmoid sinuses (52.9%) were involved, with frequent (70.6%) obstructions of multiple sinuses.^40^ Venous stroke onset is chronic for the majority of SLE patients.^41^ Venous stroke caused by SLE is indistinguishable from other etiologies in terms of neurologic symptoms.^42^ Of the 1144 venous stroke patients from the VENOST cohort, headache (73.3%) was the most common neurological symptom, followed by visual field defects (40.0%), altered consciousness (26.7%), nausea and/or vomiting (20.0%), epileptic seizures (20.0%), cranial nerve palsies (13.3%), and focal neurological deficits (6.7%).^41^

For SLE patients with venous stroke, the underlying cause of venous stroke is SLE. According to the current cases, these patients had highly active SLE and rapid disease progression, necessitating aggressive treatments to control SLE.^43^, ^44^ Venous stroke in SLE patients requires long‐term anticoagulation and immunosuppression. Glucocorticoid combined with cyclophosphamide is the preferred treatment.^40^ According to the VENOST study, complete or partial recovery was observed in most SLE patients with venous stroke; on the modified Rankin scale, 90.9% scored 0–1 after one year.^41^ SLE patients with venous stroke can experience severe neurological outcomes and should be monitored.^44^

### 2.3 Venous stroke in patients with antiphospholipid syndrome

Antiphospholipid syndrome (APS) is a systemic and noninflammatory autoimmune disease characterized by arterial and venous thrombosis and circulating antiphospholipid antibodies.^45^ APS is generally secondary to SLE but can also occur idiopathically. APS patients with venous stroke as the initial presentation is rare.^46^ The mechanism is still not clear, but it has been speculated that antiphospholipids activate endothelial cells, platelets, and monocytes, promoting the prethrombotic state.^47^ Furthermore, anti‐β2GP‐1 antibodies and complement activation play an essential role in thromboembolism.^47^ In the ISCVT study of 624 patients, 5.9% had APS as the underlying cause of venous stroke.^10^ Venous stroke is a rare manifestation in APS patients. Studies published in the literature are scarce and primarily comprise case reports or short case series; thus, there is a paucity of extensive epidemiological studies on venous stroke in APS patients.

Jerez‐Lienas et al.^15^ indicated, in a series of 27 patients and a review of the literature, that venous stroke was located in the transverse and sigmoid sinuses in 73% of patients, superior sagittal sinus in 55%, straight sinus and deep venous system in 10% and cortical veins in 14%. Sixty percent of patients had extensive thrombosis affecting multiple sinuses.^15^ APS with venous stroke as the earliest presentation was initially described in 2008.^48^ The predominant forms of clinical presentation are acute and subacute, with headaches being the most prevalent symptom reported in 74% of published cases.^15^, ^49^

In a single‐center study of the management of venous stroke patients with APS by Shen et al.,^50^ local endovascular thrombolysis, endovascular mechanical thrombectomy, and internal jugular vein stenting were utilized in patients who suffered from anticoagulant inefficacy, with satisfying outcomes. Glucocorticoids and intravenous immunoglobulin and/or plasma exchange therapy are applied in catastrophic APS‐associated venous stroke, which is characterized by microvascular thrombosis resulting in multiorgan failure.^51^, ^52^ Upon hospital discharge, 78% of patients had a complete resolution of the symptoms or mild symptoms (mRS = 0–1), 4% of patients recovered partially and maintained autonomy (mRS = 2), 10% of patients sustained mild to moderate impairments (mRS = 3–5), and 6% of patients died during hospitalization (mRS = 6).^15^ During 6 and 12 months of follow‐up, the mRS remained favorable, with the remaining symptoms disappearing.^15^, ^53^

### 2.4 Venous stroke in patients with Sjogren's syndrome

Sjogren's syndrome (SS) is a chronic systemic autoimmune disorder characterized by mononuclear cell infiltration in the salivary and lacrimal glands, whereby most characteristic symptoms consist of sicca syndrome, including xerostomia and dry eye‐xerophthalmia, but can involve multiple organs.^54^, ^55^ SS is occasionally accompanied by neurological disorders, and reports of SS leading to venous stroke are rare.^56^, ^57^ The pathogenetic mechanisms responsible for venous stroke involvement in patients with SS likewise remain unclear.^58^

Based on existing case reports and literature reviews, thrombosis was observed mostly in the transverse sinus in patients with SS accompanied by venous stroke.^57^, ^59^, ^60^, ^61^, ^62^ In addition, the straight sinus, vein of Galen, left middle cerebral vein, and inferior sagittal sinus can be involved.^57^ Given the heterogeneity in manifestations, the unique clinical characteristics of venous stroke in SS patients remain obscure. Immunosuppressive agents are combined with anticoagulant therapy to treat SS‐associated venous stroke.^61^ The anticoagulation therapy was administered in accordance with the international standard.^14^ However, without the credibility of large relevant clinical trials, the application of hydroxychloroquine is based on empirical data.^60^ Previous reports indicate that SS‐induced venous stroke patients have a favorable prognosis.^57^ Following 3–5 years of follow‐up, patients had no clinical or radiological relapse and fully recovered.^61^

### 2.5 Venous stroke in patients with inflammatory bowel disease

Inflammatory bowel diseases (IBDs) are distinguished by persistent inflammation of the gastrointestinal tract, the underlying cause of which remains unidentified.^63^ The incidence of venous stroke in patients with IBD is estimated to range from 0.5% to 7.5%.^64^ A retrospective review of 65 case reports, indicates a predilection for early onset in female patients and young individuals.^65^ The pathogenesis of thromboembolic events in IBD may encompass aberrations clotting, coagulation, fibrinolysis and the discharge of inflammatory mediators.^66^ Occurrence of venous stroke was found to be most prevalent in the super sagittal and transverse sinuses. However, a number of patients exhibited multiple thrombi in both in the deep cerebral and superficial cortical veins.^64^, ^65^, ^67^, ^68^, ^69^ The preponderant symptom among patients is headache, while other accompanying symptoms comprise seizures, focal signs, nausea, vomiting, weakness, paresthesias and encephalopathy.^64^, ^65^, ^67^, ^68^, ^69^

The co‐occurrence of autoimmune disease and venous stroke warrants consideration of a differential diagnosis of cerebral venous thrombosis when a patient presents with symptoms indicative of brain damage. Currently, there is no universally accepted treatment protocol for such patients, nor is there a consensus on the optimal interdisciplinary approach (Table 1).

**TABLE 1:** The epidemiology, circulating and imaging biomarkers, disease characteristics, treatment and prognosis of venous stroke in patients with autoimmune diseases.

	Epidemiology	Circulating and imaging biomarkers	Disease characteristics	Treatment	Prognosis
Venous stroke with BD	≈1%^11^	No specific circulating and imaging biomarkers	Recurrent oral and genital ulcers, articular, neurological, skin lesions, vascular and sight‐threatening ocular inflammation and intracranial hypertension syndrome	No standardized protocol for treatment. Primary disease and anticoagulant therapy and are necessary	Most had a good prognosis (mRS: 0–1), while a few had poor outcomes or even died (mRS ≥ 2).
Venous stroke with SLE	0.3%–1.3%^26^, ^40^	Antinuclear antibodies^70^	Neuropsychiatric lupus, arthritis, oromeningitis, vessel disease and intracranial hypertension syndrome		
Venous stroke with APS	≈5.9%^10^	Antiphospholipid antibody^71^	Thrombosis can occur in arteries, veins, and small vessels of any tissue or organ, abnormal pregnancy and intracranial hypertension syndrome		
Venous stroke with SS	Unknown	Antinuclear antibodies, anti‐SSA antibody, anti‐SSB antibody^72^	Sicca syndrome, including xerostomia and dry eye‐xerophthalmia, but can involve multiple organs and intracranial hypertension syndrome		
Venous stroke with IBD	0.5%–7.5%^64^	Calprotectin, CRP, Erythrocyte sedimentation rate^64^, ^73^	Chronic relapsing inflammation of the gastrointestinal tract, characterized by abdominal pain, diarrhea with mucus and blood in stool and intracranial hypertension syndrome		

Abbreviations: APS, antiphospholipid syndrome; BD, Behçet's disease; CRP, C‐reactive protein; IBD, inflammatory bowel disease; SLE, systemic lupus erythematosus; SS, Sjogren's syndrome.

## 3 VENOUS STROKE IN PATIENTS WITH BLOOD DISEASES

### 3.1 Venous stroke in patients with myeloproliferative neoplasms

Myeloproliferative neoplasms (MPNs) are a group of diseases, including polycythemia vera (PV), essential thrombocythemia (ET), and primary myelofibrosis (PMF), with an increased proliferation of one or more subtypes of myeloid cells.^74^, ^75^ PV and ET are associated with venous stroke.^76^ MPNs are also associated with the risk of venous stroke recurrence.^77^ Venous stroke occurs in approximately 1% of patients with MPNs.^78^ In a joint analysis of two large databases, 27 patients were found to be diagnosed with venous stroke in a series of 706 MPN patients, and nine venous stroke patients were analyzed in a cohort of 2143 MPN patients, suggesting an association between venous stroke and MPNs.^79^ The Janus kinase JAK2 exon 14 V617F mutation is present in 5.6% and 6.6% of venous stroke patients, more than 95% of patients with PV, and approximately 50% of those with ET.^80^, ^81^, ^82^ A European LeukemiaNet study by Martinelli et al.^78^ included 48 MPN patients with venous stroke and revealed prominent associations between venous stroke in patients with thrombophilia and JAK2V617F mutation.

A retrospective three‐center series was conducted by Gangat et al.^83^ and included 74 patients with venous stroke that was associated with MPNs, demonstrating that the transverse sinus (51%), sagittal sinus (43%), sigmoid sinus (35%), cerebral cortical vein/dural vein (15%), internal jugular veins (14%) and cavernous sinus (14%) were involved. The most common presenting symptom of venous stroke in patients with MPNs was headache (72% of patients), followed by focal neurological defects (34% of patients had sensory symptoms, visual loss, nausea, vomiting, aphasia, and dysarthria).^83^ Approximately 8% of patients had impaired consciousness, and 6% had seizures.

In treating MPN‐venous stroke, prevention of thrombosis and recurrence is one of the critical therapeutic goals; however, the optimal antithrombotic strategy and treatment duration are unknown.^84^, ^85^, ^86^ According to the European Academy of Neurology, for patients with venous stroke, oral anticoagulation is recommended for at least 6 months unless contraindicated.^87^ Direct oral anticoagulants (DOACs) have not yet been reported to be effective in patients with MPNs, but randomized controlled trials are currently underway.^82^ The prognosis is generally favorable; at a median follow‐up of 5.1 years of 74 patients with venous stroke associated with MPNs, recurrent venous stroke was reported in 4% of patients; in contrast, incidence rates for recurrent arterial and venous thrombosis excluding venous stroke and significant hemorrhage were 11%, 9%, and 14%, respectively.^83^ Recurrent non‐venous stroke thromboses were documented in the ELN study at an exceptionally high rate (42%), despite antithrombotic therapy being administered to most patients (80%).^78^ Taken together, findings regarding the increased risk of recurrent thrombosis following venous stroke require further investigation.

### 3.2 Venous stroke in patients with leukemia

Leukemias also be complicated by venous stroke.^82^ Thus far, several investigations have documented the occurrence of venous stroke in relation to acute promyelocytic leukemia (APL) and acute lymphoblastic leukemia (ALL), whereas there exists a paucity of reports pertaining to acute myeloid leukemia (AML) or chronic leukemias such as chronic myeloid leukemia and chronic lymphocytic leukemia with CVT.^88^, ^89^ However, there are currently no reported cases of chronic leukemias such as chronic myeloid leukemia and chronic lymphocytic leukemia combined with venous stroke. APL or ALL is a relevant cause of venous stroke in children and young adults.^90^, ^91^, ^92^ Cerebral infarction most commonly occurs at, or shortly after, the diagnosis of APL or ALL is venous stroke.^82^

#### 3.2.1 Venous stroke in APL patients

The occurrence of venous stroke in APL patients is extremely rare; only a few cases have been reported in the literature.^89^, ^93^, ^94^, ^95^ Treatment with all‐trans retinoic acid (ATRA) is the primary cause of venous stroke in APL patients.^96^ The pathophysiology of coagulopathy in APL is complex.^97^ In addition to several exogenous factors, the most compelling pathogenic mechanism probably involves leukemic cells,^98^ which release multiple mediators that can influence blood coagulation through the following mechanisms: (1) high expression of two procoagulant factors by APL cells, namely, tissue factor (TF), which triggers a cascade of coagulation by forming a potent procoagulant complex with factor VII and cell membrane phospholipids, and cancer procoagulant, which activates factor X directly in the absence of activated factor VII^99^; (2) deficiencies in fibrinogen and increases in fibrin degradation products and D‐dimers, which indicate hyperfibrinolysis^100^; (3) activation of the endothelium by adhering to APL cells resulting in clotting activation, leukocyte aggregation, hyperfibrinolysis, and thrombin generation^101^; and (4) extracellular trap cell death, which induces excess thrombin production, fibrin deposition, hyperfibrinolysis, and endothelial damage.^102^ The mechanism of venous stroke associated with APL has not yet been completely elucidated.

Seven previously published case reports of venous stroke associated with APL in patients aged 11–35 years indicated that patients were predominantly female (71%) and had transverse sinus thrombosis (85%); additionally, the superior sagittal sinus, straight sinus, sigmoid sinus, and internal jugular vein were involved.^89^, ^91^, ^92^, ^93^, ^95^, ^96^, ^103^ Most of these case reports described venous stroke secondary to APL, further worsening after initiating ATRA.^89^, ^91^, ^92^, ^93^, ^96^ However, venous stroke as an initial symptom of APL is extremely rare.^95^, ^103^ In general, patients present with a serious hemorrhagic tendency, anemia, and other symptoms of infection, followed by the typical presenting signs of venous stroke, including headaches, visual impairment, and focal neurological deficits.^89^, ^92^ The management of venous stroke in patients with APL remains controversial. Patients with APL who developed venous stroke during induction treatment with ATRA required early anticoagulation.^93^ Most case reports recommended heparin to prevent bleeding associated with thrombocytopenia and treatment‐related coagulopathy, especially during induction and consolidation therapy.^89^ If venous stroke is refractory to medical treatment or progresses to cerebral edema or hemorrhage, venous sinus thrombectomy may be essential.^89^ There was no recurrence of venous stroke or other complications from anticoagulation following treatment with ATRA and early anticoagulation.

#### 3.2.2 Venous stroke in ALL patients

Venous stroke is more frequent in patients with ALL than in those with other malignancies.^104^ The cumulative incidence of venous stroke in ALL patients was 1.4%–6.2%.^105^, ^106^, ^107^, ^108^ Risk factors for venous stroke in patients with ALL include treatment with high‐dose steroid regimens, asparaginase, and intrathecal methotrexate.^105^, ^106^, ^107^, ^108^, ^109^ In addition, a few cases of venous stroke have been documented, and they all developed following peripherally inserted central catheter placement in patients with ALL.^110^ Among ALL patients, venous stroke is most commonly reported after antileukemic therapy.^111^ The pathophysiology of coagulopathy in ALL complicated by venous stroke is complex. Tumor cells can synthesize procoagulant molecules, fibrinolytic molecules, and inflammatory cytokines; additionally, tumor cells, through direct or indirect interactions, induce the stimulation of platelets, endothelial cells, and monocytes/macrophages, downregulating anticoagulant properties and upregulating procoagulant properties.^112^, ^113^, ^114^ In addition to the substances released by tumor cells, the most compelling pathogenic mechanism involves the chemotherapeutic agents used in ALL therapy.^112^ Asparaginase and steroids have been proven to induce a hypercoagulable state by suppressing natural anticoagulants, particularly fibrinogen, antithrombin, and plasminogen, and increasing the F VIII/vWF complex.^115^, ^116^, ^117^, ^118^ Furthermore, steroid therapy is associated with a hypofibrinolytic state through dose‐dependent increases in plasminogen activator inhibitor 1.^119^, ^120^ Further studies are required to delineate the mechanism of venous stroke in association with ALL.

The absolute number of ALL patients with venous stroke is small; therefore, limited information is available on the clinical presentation, course, and outcome. Among 240 ALL patients in the Dutch–Belgian HOVON‐37 study, nine patients experienced venous stroke, and the median age was 33 years.^106^ The thrombus was located in the superior sagittal sinus (8/9), cortical vein (7/9), transverse sinus (3/9), sigmoid sinus (1/9), and straight sinus (1/9).^106^ Detailed imaging reports of venous stroke in ALL patients were made available for 39 patients in the Nordic Society of Pediatric Hematology and Oncology (NOPHO) ALL 2008 protocol by Skipper et al.^111^ indicating that the most frequent locations of thrombosis were the superior sagittal sinus (*n* = 33), transverse sinus (*n* = 20), straight sinus (*n* = 5) and confluence of sinuses (*n* = 4). Based on the present evidence, the overwhelming majority of patients are symptomatic.^107^ Forty‐three cases of venous stroke in ALL were included in a cohort study from the United Kingdom, in which frequent presenting manifestations were neurological dysfunction (65%), seizures (56%), and headaches (47%).^107^ In the NOPHO ALL 2008 study, Skipper et al.^111^ reported 46 patients diagnosed with venous stroke during ALL treatment, showing that patients had a median mRS = 2. The primary symptoms were headaches (*n* = 27), seizures (*n* = 17), motor difficulties (*n* = 16), consciousness disturbance (*n* = 13), nausea/vomiting (*n* = 12), and visual impairment and/or papilloedema (*n* = 4).

Venous stroke complicates therapy in 1%–2% of children and adolescents with ALL.^105^, ^121^, ^122^ The International Society on Thrombosis and Hemostasis recommends LMWH for at least 6 months after a venous stroke, with anti‐Xa levels monitored daily.^123^ If patients are not determined to be at increased risk of hemorrhage, anticoagulation should persist until the completion of chemotherapy and achievement of complete remission.^123^ In patients with severe thrombocytopenia (i.e., platelet count below 50 × 10^9^/L), DOACs may be considered in combination with platelet transfusion to keep platelet counts above 40–50 × 10^9^/L.^123^, ^124^ Mechanical thrombectomy, an endovascular procedure, is an alternative to anticoagulation in the case of severe venous stroke.^125^ Resuming asparaginase treatment might be considered after the stabilization of venous stroke, but the evidence regarding the safety of this option is minimal.^122^ Skipper et al. reported that 61% of patients had normal neurological status after a median follow‐up of 4.5 years, similar to findings in Klaassen et al., who reported that 35% of patients developed permanent disabilities after a median follow‐up of 4.9 years; in contrast, a later neurological morbidity of 12% was found by Musgrave et al.^107^, ^109^, ^111^ Further studies with a larger number of patients are required to confirm these findings.

### 3.3 Venous stroke in patients with multiple myeloma

Multiple myeloma (MM) remains an incurable hematologic malignancy with clonal plasma cell proliferation and the production of monoclonal immunoglobulins.^126^, ^127^ Among hematologic malignancies, patients with MM have an increased risk of developing thromboembolic complications, with at least 15% of patients developing venous stroke during the period of the disease.^128^, ^129^, ^130^ Several pathophysiological mechanisms may contribute to venous stroke in MM patients, including hyperviscosity, thrombogenic side effects of medications and dysregulation of plasma thrombin, microparticle‐associated TF, PAI‐1 overexpression, increased P‐selectin, and the accumulation of activated protein C resistance, which can result in hypercoagulability.^131^, ^132^, ^133^

The coexistence of venous stroke and MM is rare. The spectrum of neurologic complications of MM‐associated venous stroke and related conditions is complex, primary headaches.^126^, ^130^ Immunomodulatory drugs such as lenalidomide, thalidomide, and pomalidomide are widely used in MM and may be associated with an increased risk of venous thromboembolism.^134^, ^135^, ^136^ Consequently, thromboprophylactic therapy is mandatory, along with immunomodulatory therapy, either with aspirin for patients at low risk of thromboembolism or LMWH for those with higher risk.^137^ In recent years, DOACs have been registered for the treatment of venous thrombosis. Only a limited number of patients with MM‐associated venous thrombosis have been recruited in those trials. However, prescribing DOACs for these patients is not yet recommended.^138^, ^139^ To date, no study has focused on the clinical outcomes in MM patients with venous stroke. Based on a recent study, venous thrombosis developed in 52 MM patients, of whom 48 were administered thromboprophylactic therapy, with a median follow‐up of 9.0 months following onset, and five patients showed recurrence of venous thromboembolism.^138^ We speculate that MM patients with venous stroke can expect a positive outcome.

### 3.4 Venous stroke in patients with paroxysmal nocturnal hemoglobinuria

Paroxysmal nocturnal hemoglobinuria (PNH) is a rare clonal, not malignant, acquired disorder of hematopoietic stem cells characterized by intravascular hemolysis, potentially fatal venous thrombosis, and bone marrow failure.^140^, ^141^ According to PNH studies, venous thrombosis affects up to 40% of patients and represents the primary cause of death.^142^, ^143^ Venous stroke is documented in 2%–8% of patients with PNH.^82^ Nevertheless, it is an infrequent cause of venous stroke.^144^ In most PNH patients, the absence of a group of membrane proteins attached to the cell surface by glycosylphosphatidylinositol (GPI) anchors is caused by somatic mutations in phosphatidylinositol glycan anchor biosynthesis class A (PIGA) in hematopoietic stem cells.^145^, ^146^ These stem cells produce abnormal blood cells lacking CD55 and CD59, which are complement regulatory proteins, resulting in complement‐mediated intravascular hemolysis, which can trigger thrombosis by releasing free hemoglobin, platelet activation, nitric oxide depletion, endothelial dysfunction, and microparticles that promote coagulation.^147^, ^148^, ^149^, ^150^

Paroxysmal nocturnal hemoglobinuria patients with venous stroke have a highly variable natural history.^151^ The largest case series of 15 PNH patients with venous stroke was a multicenter study by Meppiel et al.,^142^ wherein 17 case reports of PNH were reviewed, and 72% of CVT‐PNH patients were found to be women. The superior sagittal sinus was the most commonly involved site for thrombosis (20/32), followed by the cortical vein (19/32), lateral sinus (14/32), deep venous system (4/32), and cavernous sinus (1/32).^142^ The clinical onset of venous stroke is subacute in most PNH patients.^152^, ^153^ Headache was the most common symptom (88%); other symptoms were focal deficits (40%), seizures (22%), and less frequently altered consciousness (22%) at the time of CVT diagnosis.^142^

Anticoagulation therapy is the primary treatment for venous stroke and consists of intravenous unfractionated heparin or subcutaneous LMWH.^154^ Eculizumab is recommended within 24 h of the onset of acute thrombosis to reduce the extent of the thrombus and prevent thrombotic recurrence.^155^ Thrombocytopenia requiring platelet transfusion is necessary to increase the platelet concentration to a target of beyond 30–50,000/L before anticoagulation is initiated.^82^ The outcome of venous stroke is worse in patients with PNH‐related venous stroke than in those with other causes of venous stroke, primarily due to new venous thrombotic events.^142^ The study by Meppiel et al.^142^ highlights that patients with venous stroke have inferior long‐term survival compared to patients without thrombosis and noncerebral thrombosis. However, the treatment and prognosis of venous stroke in PNH patients are debated and must be assessed in large long‐term studies.

### 3.5 Venous stroke in patients with thrombotic thrombocytopenic purpura

Thrombotic thrombocytopenic purpura (TTP) is a severe, rare, and relapsing life‐threatening thrombotic microangiopathy characterized by severe thrombocytopenia, microangiopathic hemolytic anemia, and ischemic end‐organ damage resulting from microvascular platelet‐rich thrombi.^156^, ^157^ TTP is a disorder resulting from genetic or acquired severe deficiency of plasma ADAMTS13, a metalloprotease that cleaves endothelium‐derived ultralarge von Willebrand factor (vWF), leading to unrestrained adhesion of the vWF multimers to platelets and microthrombi formation, resulting in thrombocytopenia and microangiopathic hemolytic anemia.^158^, ^159^, ^160^, ^161^ The predominant form of TTP is acquired immune‐mediated TTP.^162^ TTP is an infrequent cause of stroke among young adults.^163^

Thrombotic thrombocytopenic purpura‐associated venous stroke has rarely been reported. The treatment of TTP combined with venous stroke is mainly based on the treatment of the primary disease. As standard therapy, plasma exchange is used combined with immunosuppression with rituximab, cyclophosphamide, steroids, or vincristine.^164^, ^165^, ^166^ Caplacizumab, a humanized immunoglobulin fragment that recognizes vWF, has recently been found to reduce thromboembolic events, stroke, and death in patients with TTP following plasma exchange and immunosuppression.^167^ Aspirin is not recommended for treating patients with TTP‐associated venous stroke, as there is limited evidence of its efficacy and safety; nevertheless, some experts recommend it if platelet counts are above 50,000/μL.^168^ Regular infusions of fresh‐frozen plasma dramatically reduce the recurrence of stroke.^169^ Without severe bleeding or an invasive procedure, prophylactic platelet transfusions are not recommended for patients with TTP‐associated venous stroke, which may cause slight increases in the risk of thrombosis.^82^, ^170^

### 3.6 Venous stroke in patients with heparin‐induced thrombocytopenia

Heparin‐induced thrombocytopenia (HIT) is generally an acquired autoimmune disease that can be divided into classical and autoimmune types.^171^, ^172^ Classical HIT is a severe adverse drug reaction resulting in thrombosis and thrombocytopenia following heparin exposure 5–10 days before the onset of clinical presentation.^173^, ^174^, ^175^, ^176^, ^177^ Autoimmune HIT comprises the subtypes of spontaneous HIT, refractory HIT, and delayed‐onset HIT characterized by the absence of recent heparin exposure, thrombosis, and thrombocytopenia five or more days after heparin withdrawal and prolonged duration of thrombosis and thrombocytopenia, respectively.^178^, ^179^, ^180^ Rarely, with severe classical or autoimmune HIT, thrombosis may contribute to the development of venous stroke.^181^, ^182^ HIT results from the transient production of IgG platelet‐activating antibodies that recognize the multimolecular complexes of platelet factor 4 (PF4) bound to heparin.^183^ Nascent PF4‐HIT‐IgG complexes recruit additional heparin‐dependent HIT antibodies, forming multimolecular immune complexes and causing platelets to be activated, paradoxically, which can lead to CVT.^183^, ^184^

The development of venous stroke secondary to HIT is a rare occurrence documented in relatively few publications.^185^ The manuscripts describing 21 patients with HIT and associated venous stroke in Bauman et al.'s^171^ study indicate that the median age of the patients in the cohort was 55 years, and 90% of patients were females. The main locations for sinus occlusion were the transverse sinuses in 65% of patients, the sigmoid sinuses in 60% of patients, the superior sagittal sinus in 60% of patients, the jugular vein in 25% of patients, the straight sinus in 20% of patients, the torcula in 10% of patients and the cavernous sinus in 5% of patients.^171^ Among those who participated in these studies, 12 had headaches, eight had altered mental status, six had visual disturbances, four had motor dysfunction, and three suffered from seizures.^171^

Since HIT‐associated venous stroke has a high mortality rate, patients should be treated immediately with nonheparin anticoagulants, commonly known as direct thrombin inhibitors, such as argatroban and bivalirudin.^179^, ^181^, ^186^ Many debates remain regarding which drug is the best treatment option. Recent research by Sun et al.^187^ determined that in the treatment of HIT, there was no significant difference between argatroban and bivalirudin concerning thromboembolic complications. Recently, fondaparinux, which is now being studied as a possible HIT‐associated venous stroke treatment, has gained attention.^188^, ^189^ Nevertheless, more studies are needed to examine the efficacy of fondaparinux. Another standard treatment is intravenous immunoglobulin, which is often prescribed in combination with direct thrombin inhibitors and is especially effective in cases of autoimmune HIT‐associated venous stroke.^178^, ^190^ However, the prognosis of venous stroke in HIT patients is inferior to that in patients with other risk factors.^191^, ^192^, ^193^

Venous strokes frequently occur in individuals with hematological disorders. In the presence of clinical manifestations of encephalopathy damage, the likelihood of a venous stroke should not be disregarded, especially pertinent when there are notable elevations of d‐dimers, deficiencies in protein C or protein S, and other indications of thrombophilia (Table 2).

**TABLE 2:** The epidemiology, disease characteristics, treatment and prognosis of venous stroke in patients with blood diseases.

	Epidemiology	Disease characteristics	Treatment	Prognosis
Venous stroke with MPNs	≈1%^78^	The increased proliferation of one or more subtypes of myeloid cells	No standardized protocol for treatment. Primary disease and anticoagulant therapy and are necessary	Most had a good prognosis (mRS: 0–1), while a few had poor outcomes or even died (mRS ≥ 2)
Venous stroke with leukemia	≈1.4%–6.2%^105^, ^106^, ^107^, ^108^	Abnormal proliferation and accumulation of blasts in the bone marrow inhibit normal hematopoiesis, resulting in anemia, thrombocytopenia, and neutropenia		
Venous stroke with MM	15%^128^, ^129^, ^130^	Clonal plasma cell proliferation and the production of monoclonal immunoglobulins		
Venous stroke with PNH	2%–8%^82^	Intravascular hemolysis, potentially fatal venous thrombosis, and bone marrow failure		
Venous stroke with TTP	Unknown	Severe thrombocytopenia, microangiopathic hemolytic anemia, and ischemic end‐organ damage resulting from microvascular platelet‐rich thrombi		
Venous stroke with HIT	Unknown	The absence of recent heparin exposure, thrombosis, and thrombocytopenia five or more days after heparin withdrawal and prolonged duration of thrombosis and thrombocytopenia		

Abbreviations: HIT, heparin‐induced thrombocytopenia; MM, multiple myeloma; MPNs, myeloproliferative neoplasms; PNH, paroxysmal nocturnal hemoglobinuria; TTP, thrombotic thrombocytopenic purpura.

## 4 INFECTION AND VACCINATION ASSOCIATED WITH VENOUS STROKE

With the emergence of the COVID‐19 pandemic, infection and vaccination have been associated with an increase in venous stroke, which has been raising concerns.^194^, ^195^ Venous thromboembolic manifestations are predominant, particularly in patients with critical illnesses.^196^, ^197^

### 4.1 Infection associated with venous stroke

Infective venous stroke, although relatively rare, often results in a catastrophic outcome. Of 83 patients with CVT in one study, 20 (24.1%) had infective venous stroke, and pericranial infections, primarily sinusitis and orbital cellulitis, were most common.^198^ Fungal infection by aspergillosis and mucormycosis is associated with more severe complications.^198^ A retrospective multicenter cohort study that involved 13,500 patients with COVID‐19 reported an incidence of venous stroke of 8.8 per 10,000 over 3 months.^199^ Symptoms of COVID‐19 infection are often observed in patients with concomitant SARS‐CoV‐2 infection and venous stroke.^200^ In a New York metropolitan cohort study, 13,500 patients with COVID‐19 were enrolled, and 12 patients had venous stroke, with headache being the most common neurological symptom in 85% of patients, focal symptoms such as seizures and hemiparesis were present in 42% of patients, and cortical signs such as aphasia, neglect, or hemianopia were present in 25% of patients.^199^ Anticoagulation therapy and proper hydration are the mainstays of treatment for COVID‐19.^201^ Most patients received LMWH initially.^202^ The Society of Neurointerventional Surgery recommended that patients with clinical deterioration or severe neurological deficits or comas may benefit from endovascular therapy.^203^ Combined COVID‐19 infection and CVT seem to be associated with a poorer prognosis than either condition alone.^204^ Tu et al.^205^ found an excessively high mortality rate of 45.5% in COVID‐19 patients diagnosed with venous stroke. Overall, early diagnosis and prompt treatment could significantly affect the outcome of COVID‐19‐associated venous stroke patients.

### 4.2 Vaccination associated with venous stroke

DNA adenovirus vector vaccines, ChAdOx1 nCOV‐19 (AstraZeneca), and Ad26. COV2·S Johnson and Johnson (Janssen/J&J), have attracted considerable attention due to the occurrence of venous stroke with vaccine‐induced immune thrombotic thrombocytopenia (VITT).^206^ Epidemiological evidence indicates that among people who received ChAdOx1 nCOV‐19 (AstraZeneca) vaccines, the incidence rate of venous stroke was 2.6 cases per million people in the 4 months of administration.^207^ In an analysis of 155 cases of venous stroke following COVID‐19 vaccination by Gregorio et al.,^208^ multiple systemic venous thromboses were observed in 91% of patients, and thrombi occurred in almost all the main cerebral venous channels. In a cohort study by Sanchez van Kammen et al.^209^ of 116 patients with venous stroke within 28 days of SARS‐CoV‐2 vaccination, 78 presented with thrombocytopenia. Headache was the most frequent presenting symptom in patients with COVID‐19 VITT in venous stroke.^210^ Sriwastava et al.^211^ observed 14 cases of venous stroke post‐COVID‐19 vaccination; 80% presented with headache, 46% with hemiparesis, 33% with visual symptoms, and 27% with seizures. In addition, other symptoms, such as shortness of breath, malaise, vomiting, lethargy, petechiae, loss of consciousness, blurred vision, hemiparesis, abdominal pain, leg or arm weakness, and back pain, were observed in some patients.^212^ Generally, the platelet count at the time of hospital admission is approximately 50,000/L, fibrinogen levels are low, and D‐dimer levels are usually quite elevated.^213^ Testing for the presence of anti‐PF4 antibodies should not be ignored.^214^

The treatment of COVID‐19 VITT is centered on three key goals.^215^ First, thrombosis must be prevented by anticoagulation. Anticoagulation with nonheparin anticoagulants is the recommended treatment. Profound thrombocytopenia is not in conflict with anticoagulation.^216^ Patients with COVID‐19 VITT experiencing thrombotic complications are generally recommended a three‐month course of therapy. If thrombocytopenia persists, further anticoagulation may be necessary, although the patient should be re‐evaluated for thrombocytopenia.^213^ Second, autoimmune process modulation is required to prevent the activation of further platelets. Intravenous immunoglobulins should be administered in conjunction with nonheparin anticoagulants, owing to improved platelet count and recovery in COVID‐19 VITT by inhibiting serum‐induced platelet activation.^217^, ^218^, ^219^ George et al.^220^ described the successful treatment of a patient with COVID‐19 VITT and venous stroke using steroids, intravenous immunoglobulins, and bivalirudin. Patients with severe thrombocytopenia refractory to anticoagulation and intravenous immunoglobulin therapy may benefit from plasma exchange.^221^ Therapeutic plasma exchange is an extracorporeal therapy that is not currently recommended for routine use for the treatment of COVID‐19 VITT.^212^ Third, COVID‐19 VITT‐specific complications should be addressed. One of the most potentially deadly complications of COVID‐19 VITT is major bleeding, particularly intracranial bleeding. Therefore, a transfusion to correct hypofibrinogenemia should be administered to patients with fibrinogen levels below 1.5 g/L.^215^ Furthermore, decompressive craniectomy, anticonvulsant medications, or other interventions may be required in patients with extensive cerebral venous sinus thrombosis. A high mortality rate has been observed in venous stroke patients meeting the criteria for thrombocytopenia syndrome after COVID‐19 vaccination.^209^ Pavord et al.^222^ conducted a prospective cohort study of 294 patients with VITT, and the mortality was 22%.^222^ The odds of death increased by 2.7 (95% CI, 1.4 to 5.2) among those with venous stroke. In a systematic review by Waqar et al.,^223^ 160 cases from 16 countries were assessed, and the mortality rate was 36.2%. We should highlight the importance of further studies to improve recognition and determine the optimal treatment of venous stroke‐COVID‐19 VITT.

Venous stroke is a rare but serious complication that can arised from both infection and vaccination. Infections in the craniofacial region, as well as vaccination, have the potential to cause venous stroke, which cannot be disregarded.

## 5 CONCLUSION

As a particular type of stroke, venous stroke is usually regarded with favorable outcomes and is primarily prevalent among young and middle‐aged patients. However, some important risk factors, such as autoimmune diseases, hematological disorders., and even COVID‐19, are nonnegligible for venous stroke, while the specific pathologies of particular risk factors associated with venous stroke have yet to be studied. Clinical manifestations of venous stroke that cannot be explained by conventional etiologies should be evaluated for the presence of these risk factors. Anticoagulant therapy and treatment of the primary disease should be carefully evaluated before use since there are advantages and disadvantages based on the severity and complications of the disease. Generally, special types of venous stroke should not be neglected and require accurate diagnosis and prompt treatment.

## CONFLICT OF INTEREST STATEMENT

The authors declare no conflict of interest with respect to the present review study.

## Data Availability

Data sharing not applicable to this article as no datasets were generated or analysed during the current study.
